# The facilitating effect of identical objects in visual working memory

**DOI:** 10.3389/fpsyg.2022.1092557

**Published:** 2023-01-12

**Authors:** Guofang Ren, Nan Ma, Ming Lei

**Affiliations:** ^1^Education School, Anyang Normal University, Anyang, China; ^2^Research Center of Brain and Cognitive Neuroscience, Liaoning Normal University, Dalian, China; ^3^Psychological Research and Counseling Center, Southwest Jiaotong University, Chengdu, China

**Keywords:** visual working memory, identical objects, facilitating effect, spatial bias, recall task

## Abstract

According to the associative network of memory representations proposed by embedded processes models, the links between related memory representations were automatically established, which rendered these representations more easily activated. The present study adopted color recall tasks to explore whether the memory performance of identical objects was enhanced *via* the strengthening links between them, producing facilitating effect of identical objects. In Experiment 1, the number of identical items was manipulated. The results evidenced the facilitating effect, which was positively related to the number of identical objects. Experiment 2 modulated the spatial location of identical objects, which suggested that the facilitating effect was absent when two pairs of identical objects were located diagonally. Furthermore, Experiment 3 suggested that the facilitating effect was observed for the identical items which were presented in the second and fourth quadrants, rather than the first and third quadrants. Together, these results evidenced the facilitating effect of identical objects, which, however, was affected by spatial bias.

## 1. Introduction

The visual working memory (VWM) system is widely regarded as the cornerstone of cognitive functions, which bridge the external environment and mind (Baddeley, 1992; Cowan, 1999). Previous studies show that participants can only remember 3–4 simple items in a VWM task (Luck and Vogel, 1997). Although individuals can improve the precision of VWM representation by sacrificing the amount number of stored representations (Gao et al., 2011; Machizawa et al., 2012; Ye et al., 2017, 2019; Long et al., 2020), the view that VWM is limited in its storage capacity is widely accepted (Vogel et al., 2001; Alvarez and Cavanagh, 2004). In the face of the dynamically presented visual information, the memory representations were conferred with different priorities according to the task demands during the highly complex tasks (Ericsson and Kintsch, 1995; Rose, 2020; Stokes et al., 2020). In light of embedded processes models of working memory (Cowan, 1999; Oberauer, 2002, 2005), the memory items greatly attended were retained in the region of direct access (RDA) serving for the current processing, one of which was represented in the focus of attention (FOA); that was termed as the active state. Those items that were less relevant to the immediate task were maintained in the activation region of long-term memory (aLTM), and got accessed when needed later; they were regarded to be held in the passive state (LaRocque et al., 2014; Peters et al., 2019; Li et al., 2020, 2021; Zhang et al., 2022). Regarding the associative network of memory representations, the memory representations were deemed to be retained in distinct states according to the relevance to the current task, with the “state” signaling the accessibility of a representation for ongoing cognitive processing (Olivers et al., 2011; Stokes et al., 2020; Zhang et al., 2022). The memories in the active state are characterized by most privilege and direct accessibility, while the passive memories are accessed indirectly through links to the active representation (Oberauer, 2001; Oberauer and Lange, 2009; Peters et al., 2019).

During the memory task with multiple items, individuals widely adopted the chunking strategy, which rendered the memory functioning more efficient. Chunking means the links between items are generated (Ericsson and Kintsch, 1995; Oberauer, 2002). Notably, the working memory system was primarily responsible for the links of item–item and item–context, while the strength of links relied on the relation between items (Oberauer, 2002; Oberauer and Lange, 2009; Kaiser et al., 2015; Liu et al., 2022). Accordingly, we might suppose that these items with identical information tended to build up strengthening links relative to the unidentical items. When one item was activated, its linked item could be automatically activated *via* the associative links (Oberauer, 2005).

According to the embedded process models, it was proposed that the memory items were automatically bound to their positions in RDA, and the links between related memory items were spontaneously established (Oberauer and Lange, 2009). Given that the memory items with identical information were more likely to be chunked together with the strong associative links, it was assumed that the memory representations could be readily activated by means of the associative links when the other representations that were linked to it were activated. Thus, we could reasonably reckon that these memory representations that were possessed with the same feature were conferred with a low activation threshold of achieving the activation state relative to those memory items, which greatly differed. Accordingly, activation of memory representations with the same feature could require less resources due to the low activation threshold, thereby the spared resources preferably being used to enhance the memory performance. If it was the case, we predicted that the memory precision should be enhanced when presenting more identical items, while the spatial information of memory items might play a role. Thus, that opened up the question of whether the strong associative links between identical items could contribute to better memory performance, that is, the facilitating effect of identical objects.

In this study, we attempted to explore the facilitating effect of identical objects by measuring the precision and quantity of memory representations in color recall tasks. Based on the definition of an identical object, we defined the two bars with the same orientation as the identical objects. The bar’s orientation should be remembered precisely during the encoding and retention period and retrieved later when probed. The size of the memory set did never exceed the memory capacity (no more than four items), which ensured the successful encoding of all memory items (Luck and Vogel, 1997; Cowan, 2001).

## 2. Experiment 1

We manipulated the number of identical objects to assess the facilitating effect of identical objects in Experiment 1, generating three conditions, two pairs of identical objects (2-pair), one pair of identical objects (1-pair), and no identical objects (0-pair). Considering that the size of the memory set was less than four, the memory quantity was comparable across the three conditions. We predicted that, if the facilitating effect of identical objects existed, the memory precision in the 2-pair and 1-pair conditions would be better than that of the 0-pair condition; if not, the memory precision in the three conditions did not differ.

### 2.1. Method

#### 2.1.1. Participants

There were 12 participants (six female participants; mean age: 23.67 ± 3.06 years) from the Liaoning Normal University. Each of them signed the written informed consent before the experiment and received 15 CNY after participation for compensation. They all reported right-handedness and normal color vision, as well as normal and corrected-to-normal sight. The research was authorized by the Research Ethics Committee of Liaoning Normal University.

#### 2.1.2. Stimuli

Each sample array contained four black-oriented bars (1.1° × 0.2°). The four bars were located at the four quadrants on a gray (RGB value, 120, 120, 120) screen. Each bar was in the center of each quadrant. The orientation of each bar changes between 0° and 179° at a 1° distance, producing 180 directions. All orientations of bars were randomly selected from them. The orientations of any two bars in the memory array were either identical or different. The orientations of the two different bars differed by at least 30°. The procedure was run by E-Prime 1.1 software. Visual stimuli were displayed on the 19-inch screen (60-Hz refresh rate, 1,024 × 768 pixels). Participants’ responses were recorded from the computer mouse and keyboard. There was a fixation cross (0.2°) in the middle and consistently visible until finishing the experiment.

#### 2.1.3. Procedure

**Figure 1:**
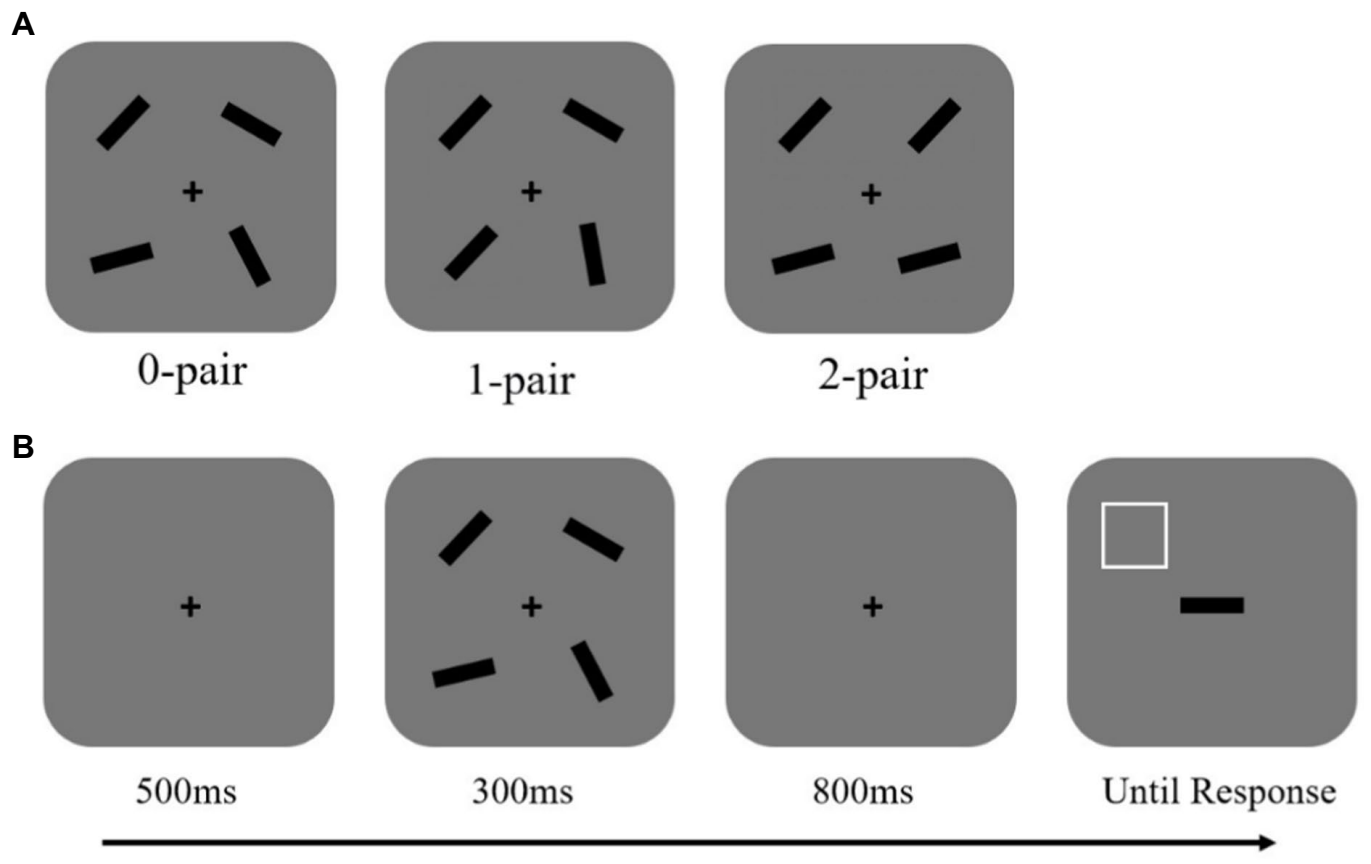
**(A)** Spatial configuration of memory items in Experiment 1. There were three conditions: 0-pair condition, 1-pair condition, and 2-pair condition. **(B)** The schematic of the experimental procedure. The white square outline was used as a cue to instruct participants to recall the orientation of the bar, which previously appeared at that cued location.

Experiment 1 was conducted by a within-subject design. The memory array consistently presented four objects. The objects were bilaterally presented because previous studies suggest that, due to the allocation of more attentional resources, VWM performance is better when visual items are allocated in both left and right visual fields than within only one hemifield (Umemoto et al., 2010; Zhang et al., 2018). There were three conditions: two pairs of identical bars (2-pair), one pair of identical bars (1-pair), and no identical bars (0-pair; see Figure 1A). The 2-pair condition meant that the orientation of two bars which might be horizontally or vertically arranged in the memory array was the same, and the directions of the other two arranged horizontally or vertically were also the same, but the orientations of the first two were different from the latter two. The 1-pair condition indicated that the orientations of two objects were identical, but the orientations of the other two were different; the orientations of the first two were different from the latter two. The 0-pair condition indicated that the four bars had completely different orientations, so there was no pair of identical objects.

Before the appearance of the memory array lasting for 300 ms, only the fixation cross was presented for 500 ms. Then the screen was blank, lasting for 800 ms, and a test display appeared. In the probe array, a horizontal bar appeared centrally, and a white square outline which indicated to recall the orientation of the bar which previously appeared at that cued location. The test display did not disappear until making a response (see Figure 1B). The horizontal bar needs to be rotated to reproduce an orientation the same as the original at that square-cued location. Participants adjusted the orientation of the bars by clicking the two mouse buttons: a button used for rotating the bar at a free angle and the other used for fine adjustment by increasing or decreasing 1° per click. When participants were satisfied with their response, the probe array disappeared, and the next trial began.

We provided participants with instructions before starting formal trials. They were informed to make a response as accurately and quickly as possible, meanwhile the accuracy was emphasized. Three conditions were mixed randomly. Each condition contained 160 trials, creating 480 trials a total. Before starting, a practice of 20 trials was carried out by participants. The whole experiment lasted approximately 1 h.

### 2.2. Result

**Figure 2:**
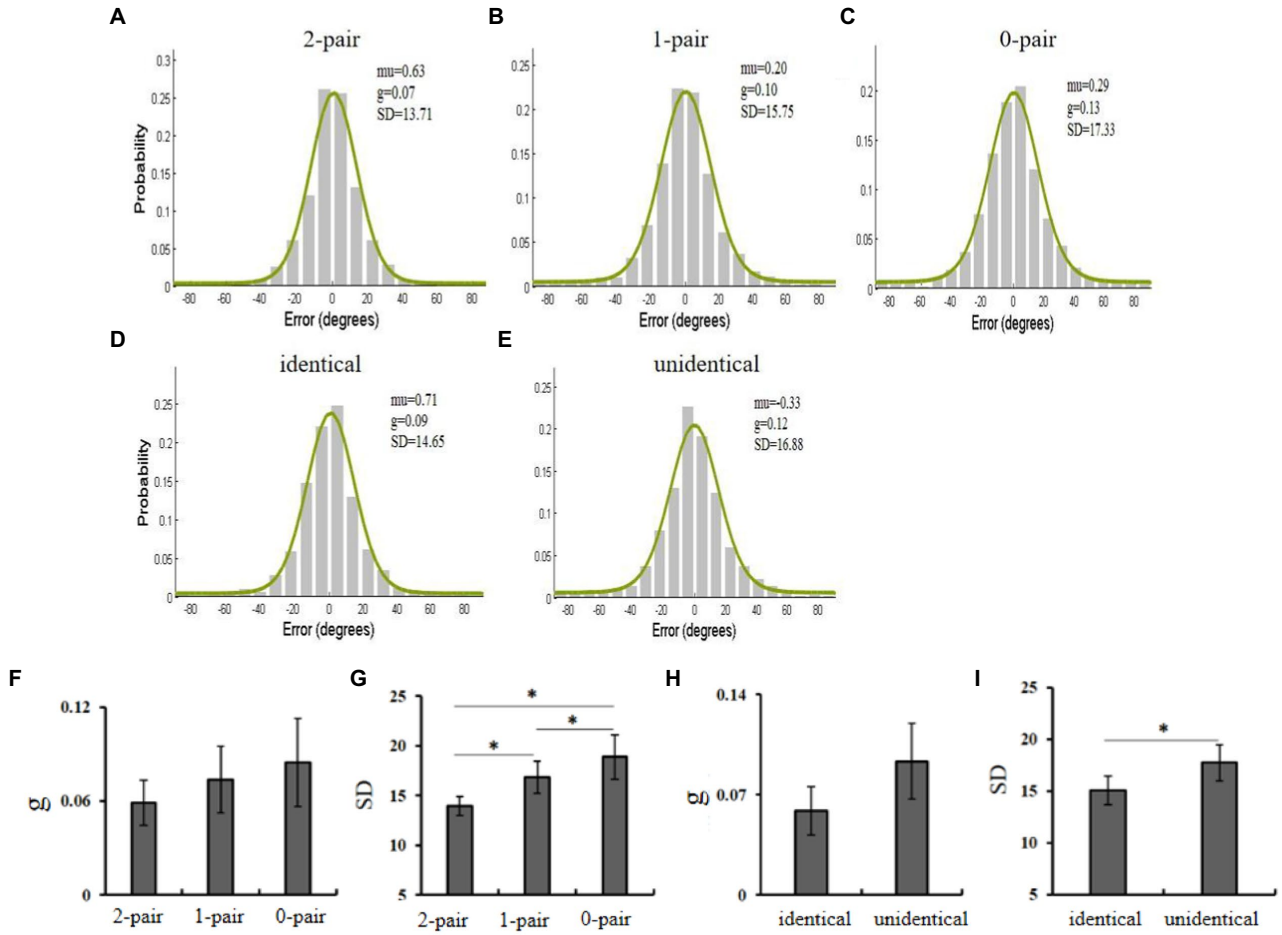
**(A–C)** Distributions of response errors with the fit of standard mixture model in the condition with two pairs of identical objects (2-pair), one pair of identical objects (1-pair), and no pair of identical objects (0-pair). **(D,E)** The distributions of response errors for the identical items and unidentical items in the 1-pair condition. **(F,G)** The guess rate and *SD* in the three conditions. **(H,I)** The guess rate and *SD* for the identical items and unidentical items in the 1-pair condition. The black bars represent the standard error.

The Mem toolbox was used for the data analysis (Suchow et al., 2013). Individuals’ data were fitted with the mixture model (Zhang and Luck, 2008). The standard deviation (*SD*) indexed the width of the distribution of memory errors, and its reciprocal could reflect the memory precision. The guess rate (*g*) indicated the height of uniform distribution, reflecting the storage probability, that is, memory quantity. Then, *SD* and *g* of all participants were averaged under the three conditions (Figures 2A–E).

The guess rate (*g*) and *SD* were subject to one-way ANOVA separately. η_p_^2^ was reported as effect size. We used Greenhouse–Geisser adjustment to correct p. There was no main effect of guess rate, *F*(2, 22) = 1.06, *p* > 0.05, η_p_^2^ = 0.09, while the significant main effect of memory precision was observed, *F*(2, 22) = 10.75, *p* < 0.05, η_p_^2^ = 0.49. *Post hoc* test suggested that the memory precision in the 2-pair condition was high, and the precision of the 1-pair was better than the 0-pair (all *p* < 0.05). These results suggested that the guess rate was independent of the number of identical objects, while the memory precision increased as more pairs of identical objects were presented, revealing the facilitating effect of identical objects (Figures 2F,G).

If the enhancement of memory precision resulted from the sameness of objects, we then expected that, in the 1-pair condition, the memory precision of identical objects was better than that of the other two objects. As we expected, the results of the paired-sample *t*-test showed that the guess rate did not differ between the identical and unidentical objects, *t*(11) = 3.98, *p* > 0.05, Cohen’s d = 0.27, whereas the memory precision of identical objects was greatly higher than unidentical objects, *t*(11) = 8.01, *p* < 0.05, Cohen’s d = 0.42 (Figures 2H,I).

### 2.3. Discussion

The earlier results indicated that the memory precision of the identical objects was better than that of the unidentical, which reflected the facilitating effect of identical objects. That is, the memory precision improved as more pairs of identical objects were remembered. For the external visual environment, the visual information could be perceptually structuralized even for the discrete items. According to the Gestalt principles, the discrete items can be encoded as an integrated object, which specifically guides attentional resources and then impacts the cognitive process (Palmer and Rock, 1994; Treisman and Zhang, 2006). According to the binding mechanisms in the RDA, the item was bound to its context automatically (Oberauer and Lange, 2009). That seemed to imply that the link strength between items might be affected by their spatial distance. In other words, the links of identical items might be weak when they are presented at a relatively far distance, while the links are relatively strong at a close distance. Considering that the facilitating effect of identical objects was observed for the memory items located horizontally or vertically in Experiment 1, that leaves up the question of whether the facilitating effect still occurred when locating the identical items diagonally. That question was explored in Experiment 2.

## 3. Experiment 2

In this part, we aimed to explore the effect of location information on the facilitating effect of identical objects. The locations of identical objects were manipulated. Based on the experimental conditions in Experiment 1, we designed the horizontal condition (2-pair-horizontal), vertical condition (2-pair-vertical), diagonal condition (2-pair-diagonal), and four unidentical conditions (4-object). There were two pairs of identical objects in each condition. In addition, there was a condition of presenting two unidentical items as baseline (2-object).

### 3.1. Method

#### 3.1.1. Participants

Recruitment of 12 undergraduates (seven female participants, mean age: 23.67 ± 1.89 years) who came from the Liaoning Normal University; they received 15 CNY for compensation for their participation. Written informed consents were signed by each participant. They reported normal color vision and normal or corrected-to-normal sight.

#### 3.1.2. Stimuli and procedure

**Figure 3:**
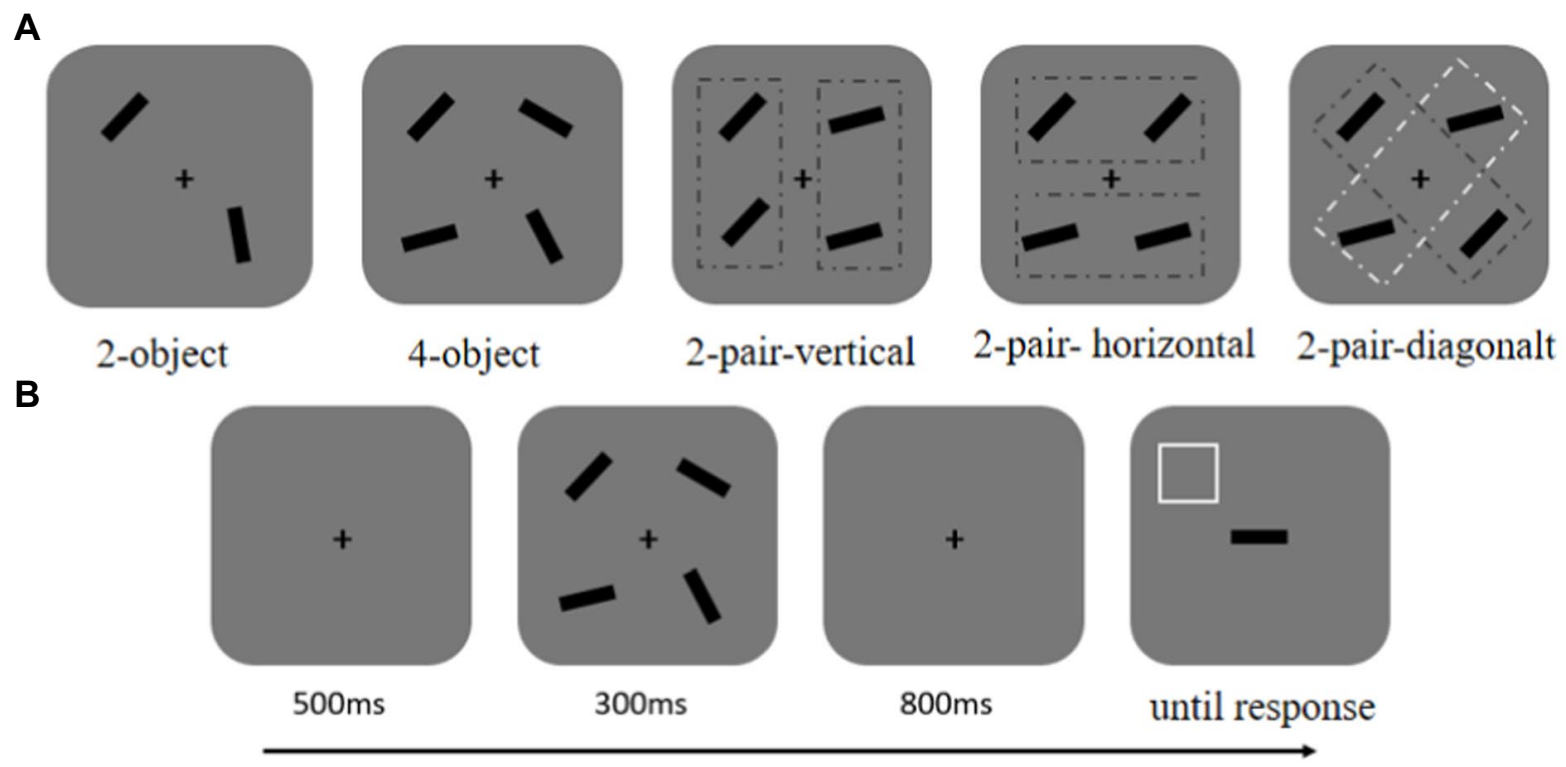
**(A)** Spatial configuration of memory items in Experiment 2. The memory arrays consisted of two unidentical objects (2-object), four unidentical objects (4-object), two pairs of identical objects arranged vertically (2-pair-vertical), two pairs of identical objects arranged horizontally (2-pair-horizontal), and two pairs of identical objects arranged diagonally (2-pair-diagonal). **(B)** The schematic of a trial.

The stimuli and procedure in this experiment were similar to the previous experiment (see Figure 3B) but involved some modification. The memory arrays consisted of two unidentical objects (2-object), four unidentical objects (4-object), two pairs of identical objects arranged horizontally (2-pair-horizontal), two pairs of identical objects arranged vertically (2-pair-vertical), and two pairs of identical objects arranged diagonally (2-pair-diagonal; see Figure 3A). Participants need to perform all the conditions. The five conditions were mixed randomly with each condition containing 80 trials. There were 400 trials across five blocks. Before the formal experiment started, participants needed to perform 20 trials for practice. That experiment lasted approximately 1 h.

### 3.2. Result

**Figure 4:**
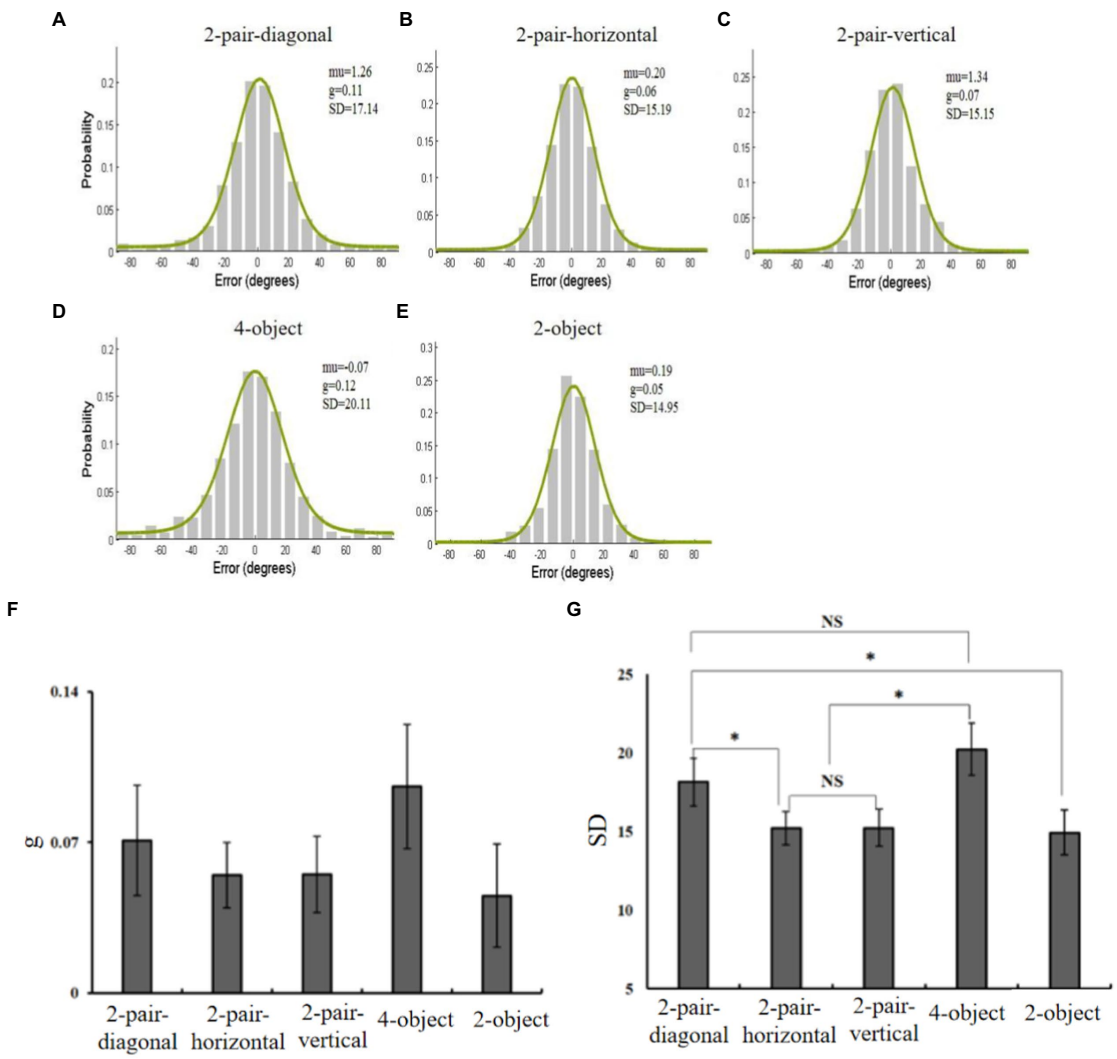
**(A–E)** Distributions of response errors with the fit of standard mixture model in the 2-pair-diagonal, 2-pair-horizontal, 2-pair-vertical, 2-object, and 4-object conditions. **(F,G)** The guess rate and *SD* in the five conditions. The black bars represent the standard error.

The offsets between the response and original values were fitted using mixture models, generating the guess rate (*g*) and *SD* (Figures 4A–E). The guess rate and *SD* were separately analyzed by one-way ANOVA. The results showed no main effect of guess rate, *F*(4, 44) = 1.57, *p* > 0.05, η_p_^2^ = 0.13, while the main effect of *SD* was significant, *F*(2.5, 28) = 8.58, *p* < 0.05, η_p_^2^ = 0.44 (Figures 4F,G). *Post hoc* analysis suggested that the precision of 2-pair-diagonal items was greatly lower than 2-object, 2-pair-horizontal, and 2-pair-vertical (all *p* < 0.05), while comparable to the 4-object (*p* > 0.05). The precision did not differ between the 2-pair-horizontal and the 2-pair-vertical conditions (*p* > 0.05). The 2-object had statistically better precision than the 4-object (*p* < 0.05). These results are depicted in Figures 4F,G, demonstrating that the spatial position of identical objects had an effect on the facilitating effect.

### 3.3. Discussion

These results suggested that the identical objects were conferred with higher memory precision compared to the unidentical objects when the identical items were located horizontally and vertically, manifesting the facilitating effect again. Importantly, the facilitating effect was absent when the identical items were presented in a diagonal manner. The absence of facilitating effect might be explained by the fact that the associative links between the two pairs of identical items intersected due to the diagonal position of the two pairs, which thus caused the interference and then overrode the facilitating effect. If this was the case, it should be expected a facilitating effect when a pair of identical objects was presented diagonally, which was explored in the following part.

## 4. Experiment 3

### 4.1. Method

#### 4.1.1. Participants

Notably, 14 undergraduates (13 female participants, mean age: 21.43 ± 2.13 years) took part in Experiment 3 and received 15 CNY after the completion of the experiment. Participants provided written informed consent when they arrived at the lab. All reported right-handedness and normal color vision, as well as the normal or corrected-to-normal sight.

#### 4.1.2. Stimuli and procedure

**Figure 5:**
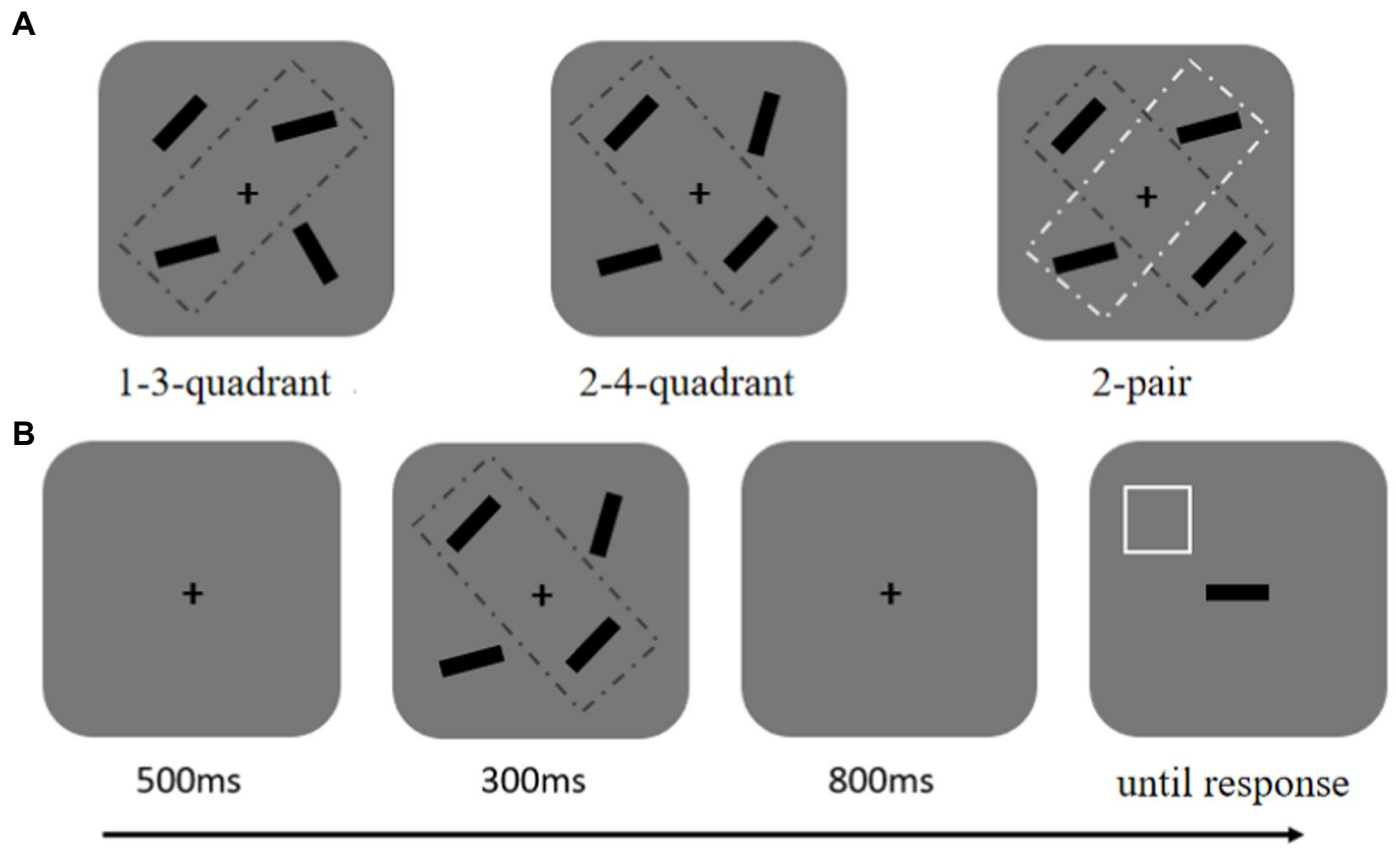
**(A)** Spatial configuration of memory items in Experiment 3. **(B)** The schematic of a trial.

We adopted similar stimuli and procedure to the Experiment 1 in this part, excepting the following (see Figure 5B). The color recall task was conducted in a within-subject design. There were constantly four items in each condition, and the identical objects were always presented in diagonal. That generated three conditions, one pair in the first and third quadrants (1-3-quadrant condition), one pair in the second and fourth quadrants (2-4-quadrant condition), and two pairs in diagonal (2-pair condition; see Figure 5A). The three conditions were mixed randomly; each condition contained 80 trials with five blocks. At least 1 min was inserted between blocks for a rest. Before the formal trials, participants received 20 trials for practice, ensuring that to be well familiar with the experimental procedure.

### 4.2. Results

**Figure 6:**
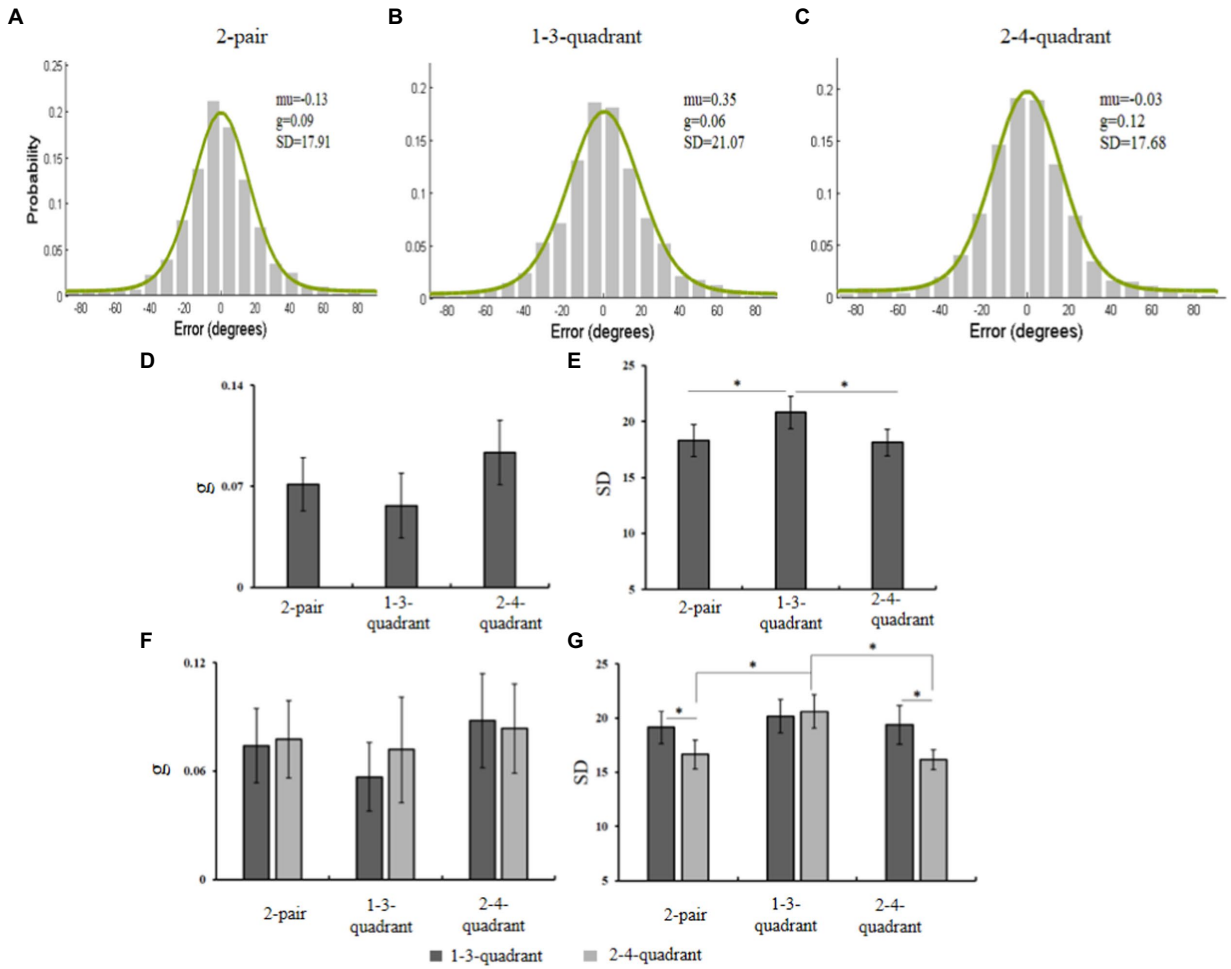
**(A–C)** Distributions of response errors with the fit of standard mixture model in the 2-pair, 1-3-quadrant, and 2-4-quadrant conditions. **(D,E)** The guess rate and SD in the three conditions. **(F,G)** The guess rate and SD of items in the 1-3-quadrant and 2-4-quadrant under the three conditions. The black bars represent the standard error.

The offset between response and original values was fitted using the mixture model (Figures 6A–C), producing *SD* and guess rate (*g*). We run a one-way ANOVA on them separately. The results showed that the main effect of guess rate was not significant, *F*(2, 26) = 2.26, *p* > 0.05, η_p_^2^ = 0.15, but a significant main effect of memory precision was observed, *F*(2, 26) = 8.28, *p* < 0.05, η_p_^2^ = 0.39. A subsequent simple effect test showed that the precision in the 2-pair condition was higher than the 1-3-quadrant condition (*p* < 0.05), and the precision in the 2-4-quadrant was also better than the 1-3-quadrant condition (*p* < 0.05). The precision of the 2-pair matched the precision of the 2-4-quadrant (*p* > 0.05).

To explore the effect of diagonal location on the facilitating effect, we then conducted a 2 (quadrant:1–3 vs. 2–4) × 3 (location of identical items: 2-pair vs. 1-3-quadrant vs. 2-4-quadrant) ANOVA on *SD* and *g* (Figures 6D,E). For the guess rate, there was no main effect of the location of identical items, *F*(2, 26) = 1.03, *p* > 0.05, η_p_^2^ = 0.07, neither nor the main effect of the quadrant, *F*(1, 13) = 0.11, *p* > 0.05, η_p_^2^ = 0.01. The two factors did not significantly interact, *F*(2, 26) = 0.14, *p* > 0.05, η_p_^2^ = 0.01. For the memory precision, the main effect of quadrant was not significant, *F*(1, 13) = 4.13, *p* > 0.05, η_p_^2^ = 0.24, neither was the main effect of the location of identical items, *F*(2, 26) = 2.62, *p* > 0.05, η_p_^2^ = 0.17. Importantly, the interaction was significant, *F*(2, 26) = 7.00, *p* < 0.05, η_p_^2^ = 0.35. The subsequent analysis showed that, for the items in the first and third quadrants, the memory precision was comparable across the three conditions. Whereas for the items in the second and fourth quadrants, the memory precision in the 1-3-quadrant condition was significantly lower than the 2-4-quadrant and 2-pair conditions (*p* < 0.05), and the latter two matched with each other (*p* > 0.05). In the 2-pair condition, the memory precision of objects in the second and fourth quadrants was higher than the other two (*p* < 0.05); in the 2-4-quadrant condition, the precision of objects in the second and fourth quadrants was higher than others (*p* < 0.05); in the 1-3-quadrant condition, the four objects have comparable precision (*p* > 0.05). These results showed that the objects in the second and fourth quadrants have an advantage in behavioral performance over those in the first and third quadrants. These results are depicted in Figures 6F,G.

### 4.3. Discussion

These results showed that the identical items that were located in the first and third quadrants were not endowed with facilitating effects, whereas the facilitating effect still occurred when they were located in the second and fourth quadrants. Therefore, these results revealed that the facilitating effect of identical items was greatly related to the quadrant information when they were located diagonally, which might be interpreted by spatial bias.

## 5. General discussion

This current study attempted to explore whether the memory performance of identical objects could be enhanced due to the strengthening links between them across three experiments. We varied the number of identical items in Experiment 1, and these results showed that the memory precision was higher when more identical items were presented, indicating the facilitating effect of identical objects. Given that the memory items were automatically bound with spatial context according to the binding mechanisms (Oberauer, 2002; Oberauer and Lange, 2009), the location information of memory items should be taken into account. In addition to the horizontal and vertical conditions, two pairs of identical items were located diagonally in Experiment 2. We observed the absence of facilitating effect of identical items. However, it was premature to consider that the facilitating effect completely relied on space proximity (Shepard, 1962). Furthermore, Experiment 3 examined what the effect the spatial location on the facilitating effect. In this part, the identical items were always presented diagonally and differed in the quadrant. It was found that the identical items in the second and fourth quadrants still conferred with facilitating effect, but rather in the first and third quadrants. Overall, we concluded that the object sameness contributed to better memory performance, evidencing the facilitating effect of identical objects; that facilitating effect was conditioned by the spatial context, thus not extended to the identical objects that were presented in any random positions.

In terms of the memory system, the associative network of memory representations was established *via* the abundant links between related representations. The high relevance between the representations preferentially contributed to strengthening links. In the current study, the memory precision acted as the behavioral indicator of the linking strength of memory representations, which to some certain revealed that physically identical objects produced relatively stronger links than unidentical objects. Notably, the linking strength was conditioned by location information, which at least complied with the proximity of the Gestalt principle (Peterson and Berryhill, 2013; Shen et al., 2014).

Moreover, it has previously been proposed that spatial information plays an important role. When visual stimuli appeared within the field of vision, individuals would involuntarily focus on a specific visual field, which resulted in the preferable processing of the stimuli in the focused visual field over other unfocused visual fields. That was referred to as spatial bias (Ossandon et al., 2014). The facilitating effect from the identical objects located in the second and fourth quadrants might be accounted for by the spatial bias that was thought to derive from the interaction between sensory attention and motor intentional (Schwartz et al., 1997). For example, a previous study has found that the left visual field bias worked during the early perception of face stimuli. That bias was presumed to be accounted for by the hemisphere lateralization or the product of control by high-lever brain areas. In addition, the social culture might also have an effect on spatial bias. Brady et al. (2005) found that, for example, people tended to depend on the expressions of the left face for making a response when the left and right faces wore different expressions during a face discrimination task; however, the left bias was absent for those people who habitually wrote and read from right to left.

The research on spatial bias previously suggested that participants showed a leftward bias in the early period of visual research, independent of the stimuli category (Ossandon et al., 2014). In addition, a study on the gaze bias had revealed that individuals tended to gaze at the upper-left location in the initial gaze movement when they performed a visual search; in addition, the upper-left and lower-right parts were thought as favored locations, though depending on conditions (Durgin et al., 2008). That is, we are more likely to focus on the top part when the attention shifted to the left visual field, while the focus was put on the down part when attending to the right visual field. Intriguingly, this research on the spatial bias convergingly pointed to the second or/and fourth quadrants, which was compatible with the findings of the current study. Though there was no agreement on the quadrant edge, it seemed to presume that the second quadrant was widely regarded to be the most prioritized.

The sameness of memory items should be regarded as the extremity of the similarity of memory items. There were two theoretical interpretations that might underpin the facilitating effect of similarity. First, it has been pointed out that the enhanced memory performance resulted from the simplification of the memory array due to the similarity of memory items, thus indirectly reducing the memory load and lowering the need for cognitive resources (Mate and Baques, 2009). However, Lin and Luck (2009) argued that better memory performance of similar items was attributed to reciprocal facilitation (Lin and Luck, 2009). The current results pattern suggested that the guess rate was low and comparable across different conditions, indicating that the number of memory items successfully encoded in VWM did not differ. That thus denied the reducing memory load account. In addition, considering that the memory precision should reach an asymptote when the load reached four, intriguingly, the memory precision was modulated by the number of identical items through which four items were remembered, which implied the reciprocal facilitation between the identical items *via* strengthening links. That seemed to be consistent with Lin’s proposal.

## 6. Conclusion

Overall, the current study evidenced the facilitating effect of identical objects, while the facilitating effect was affected by location information (i.e., spatial bias). In further research, it was necessary to investigate what the role of spatial distance and configuration in the facilitating effect of identical objects. Given that the facilitating effect was observed from two identical objects in the current study, the present findings paved the way for further exploration of whether the facilitating effect was larger if the number of identical objects was more than two, and whether the facilitating effect had a similar level when increasing the overall memory load. Future research on these questions would benefit from a complete understanding of the maintenance mechanisms of identical items.

## Data availability statement

The raw data supporting the conclusions of this article will be made available by the authors, without undue reservation.

## Ethics statement

The studies involving human participants were reviewed and approved by Research Ethics Committee of Liaoning Normal University. The patients/participants provided their written informed consent to participate in this study.

## Author contributions

GR and NM developed the study concept and designed the experiment, performed the data analysis, and provided critical revision. NM performed testing and data collection. GR, NM, and ML interpreted the data. ML drafted the manuscript and refined the language. All authors contributed to the article and approved the submitted version.

## Conflict of interest

The authors declare that the research was conducted in the absence of any commercial or financial relationships that could be construed as a potential conflict of interest.

## Publisher’s note

All claims expressed in this article are solely those of the authors and do not necessarily represent those of their affiliated organizations, or those of the publisher, the editors and the reviewers. Any product that may be evaluated in this article, or claim that may be made by its manufacturer, is not guaranteed or endorsed by the publisher.
